# *SMIM1* variants rs1175550 and rs143702418 independently modulate Vel blood group antigen expression

**DOI:** 10.1038/srep40451

**Published:** 2017-01-13

**Authors:** Mikael K. Christophersen, Magnus Jöud, Ram Ajore, Sunitha Vege, Klara W. Ljungdahl, Connie M. Westhoff, Martin L. Olsson, Jill R. Storry, Björn Nilsson

**Affiliations:** 1 Division of Hematology and Transfusion Medicine, Department of Laboratory Medicine, Lund University, Lund, Sweden; 2 Clinical Immunology and Transfusion Medicine, Laboratory Medicine, Office of Medical Services, Lund, Sweden; 3 Laboratory of Immunohematology and Genomics, New York Blood Center, New York City, NY, USA

## Abstract

The Vel blood group antigen is expressed on the red blood cells of most individuals. Recently, we described that homozygosity for inactivating mutations in *SMIM1* defines the rare Vel-negative phenotype. Still, Vel-positive individuals show great variability in Vel antigen expression, creating a risk for Vel blood typing errors and transfusion reactions. We fine-mapped the regulatory region located in *SMIM1* intron 2 in Swedish blood donors, and observed a strong correlation between expression and rs1175550 as well as with a previously unreported tri-nucleotide insertion (rs143702418; C > CGCA). While the two variants are tightly linked in Caucasians, we separated their effects in African Americans, and found that rs1175550G and to a lesser extent rs143702418C independently increase *SMIM1* and Vel antigen expression. Gel shift and luciferase assays indicate that both variants are transcriptionally active, and we identified binding of the transcription factor TAL1 as a potential mediator of the increased expression associated with rs1175550G. Our results provide insight into the regulatory logic of Vel antigen expression, and extend the set of markers for genetic Vel blood group typing.

Recently, we and two other research groups identified a previously unreported erythroid gene, Small Integral Membrane Protein 1 (*SMIM1*), as the locus of the Vel blood group system^1^,^2^,^3^. We showed that individuals who lack the Vel blood group antigen (about 1/1,200 in Sweden and 1/4,000 globally) are homozygous for an inactivating 17-bp deletion in *SMIM1* exon 3.

While these findings provide a molecular background for the Vel-negative (Vel−) phenotype, they do not explain the recurrent clinical observation that Vel-positive (Vel+) individuals show great variability in antigen expression. Weakly Vel+ erythrocytes (Vel+^weak^) can be mistyped as Vel− by routine serological phenotyping, creating risk for transfusion reactions in patients with anti-Vel inadvertently transfused with Vel+^weak^ erythrocytes. While some of the variation can be attributed to heterozygosity for the 17-bp deletion^1^, this mutation does not account for all the variation. The common SNP rs1175550 (A > G, minor allele frequency, MAF = 0.22 in Caucasians), located in intron 2 of *SMIM1*, associates with mean corpuscular hemoglobin concentration (MCHC)^4^, and individuals who carry the minor allele (rs1175550G) express *SMIM1* at a higher level than individuals who carry the major allele (rs1175550A)^2^,^5^,^6^. However, it is not known whether rs1175550 itself is causal, or a proxy for another (as yet undetected) causal variant. Moreover, the molecular effects of this variant have not been defined, or whether additional variants modulate *SMIM1* expression.

To address these questions, we fine-mapped a candidate regulatory region in *SMIM1* intron 2 containing rs1175550 and multiple erythroid transcription factor binding sites in Swedish and African American blood donors. We identified rs1175550 and a previously unreported tri-nucleotide insertion (rs143702418; C > CGCA) as correlated with *SMIM1* and Vel antigen expression. While these variants, and their effects on expression, were inseparable in the Swedish samples, their effects could be separated in the African American samples where the linkage disequilibrium in *SMIM1* intron 2 was not as tight. Our data show that rs1175550 and rs143702418 independently modulate *SMIM1* expression, although the effect of rs1175550 dominated that of rs143702418. Using gel shift assays, we found that both variants influence nuclear protein binding, and that the strong effect of rs1175550 may be mediated by differential binding of TAL1.

## Results

### rs1175550 is located in a regulatory region in SMIM1 intron 2

**Figure 1:**
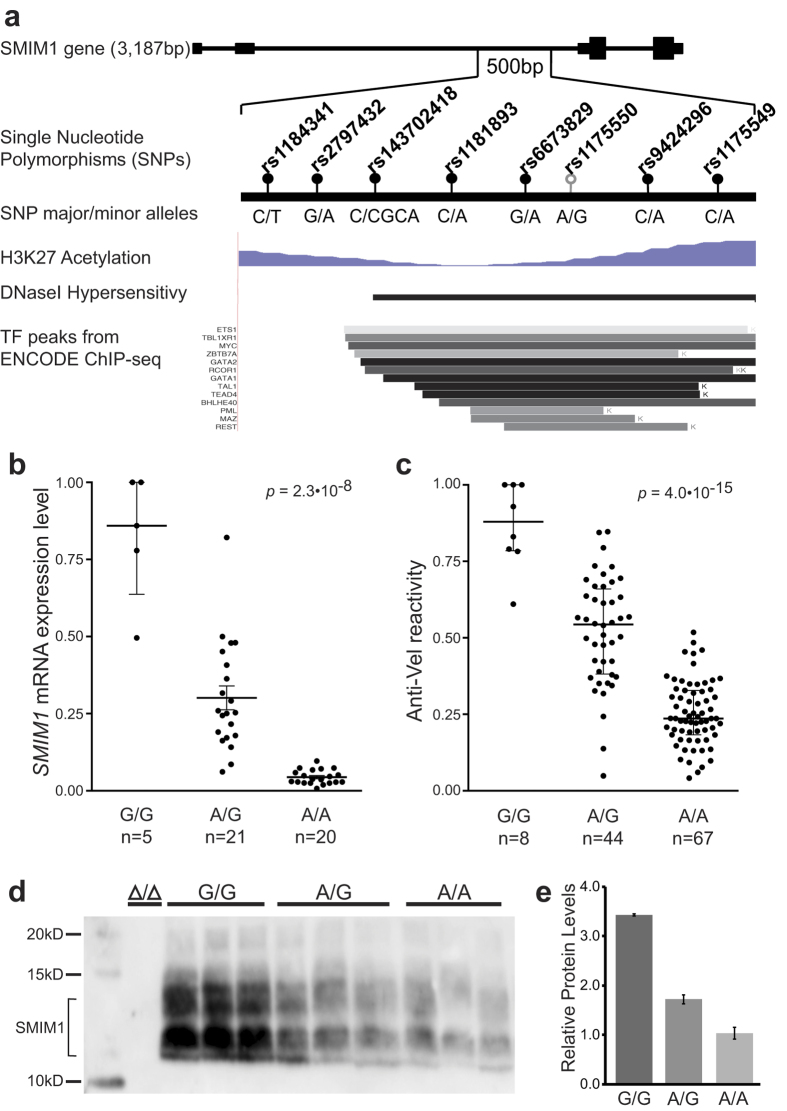
rs1175550 and rs143702148 association with *SMIM1* and Vel antigen expression. **(a)** Diagrammatic representation of the region around rs1175550 with neighbouring variants and data from the UCSC Genome Browser. Blue: ENCODE H3K27 ChIP-seq data for K562 erythroleukemia cells. Black: ENCODE DNaseI hypersensitivity data. Bottom: Transcription factor binding sites inferred by ChIP-seq in the ENCODE project; greyscale indicates signal strength, “K” indicates that the data was produced in K562 cells. Transcription factors with strong signal or known function in erythropoiesis were included. (**b**) *SMIM1* expression in the Swedish samples by RT-qPCR. Expression values were normalised against the sample with the highest expression and plotted individually with indication of the median and inter-quartile range (IQR). Statistical analyses were performed with Kruskal-Wallis one-way analysis of variance with Dunn’s multiple comparisons test. (**c**) Vel antigen expression on RBCs measured by flow cytometry (MFI; median fluorescence intensity) with human anti-Vel serum and PE-conjugated goat-anti-human secondary antibody. All samples were normalised to the sample with the highest expression and plotted together with the median and IQR. Statistical analyses were performed with Kruskal-Wallis one-way analysis of variance with Dunn’s multiple comparisons test. Association data for the seven other SNPs, including rs143702418, in the region are given in Supplementary Figs S1 and S2. (**d**) Western blot with polyclonal anti-SMIM1 on a subset of 9 samples from the Swedish collection with the indicated rs1175550 genotype plus a control sample homozygous for the 17-bp deletion. SMIM1 is thought to exist as a monomer and dimer in the RBC membrane. (**e)** Bands in D) quantified by densitometry analysis. Mean values ± standard error of the mean after correction for total protein, n = 3 for each genotype.

To understand the role of rs1175550, we examined its neighborhood in *SMIM1* intron 2 for transcription factor binding sites using ChIP-seq data in the Encyclopedia of DNA Elements compendium (ENCODE)^7^. This revealed a 500 bp region with increased acetylation of lysine 27 on histone 3, increased DNaseI hypersensitivity, and binding sites for multiple transcription factors, including the erythroid factors GATA-1, TAL1 and ZBTB7A. The region contained eight sequence variants annotated with a MAF of more than 1% in dbSNP 144^8^ (Fig. 1a).

### rs1175550 and rs143702418 associate with SMIM1 and Vel expression

To test for correlations with *SMIM1* and Vel expression, we sequenced the identified region in 150 Vel+ Swedish blood donors. In the same individuals, we quantified *SMIM1* and Vel antigen expression by quantitative PCR and flow cytometry. We detected all eight candidate sequence variants at frequencies similar to those reported in the 1000 Genomes catalog, phase 3^9^ containing fully sequenced genomes from 2,504 individuals grouped into five superpopulations (African, American, East Asian, European and South Asian) (Table 1). The rs1175550 variant was strongly associated with *SMIM1* mRNA levels (*p* = 2.1∙10^−8^), Vel antigen expression (*p* = 4.0∙10^−15^) and SMIM1 protein on erythrocyte membranes, with the G allele correlated with higher expression (Fig. 1b–d). Interestingly, we found almost identical allele frequencies and correlation values for a previously unreported C > CGCA insertion rs143702418 (rs70940313 in reverse complement), located only 96 basepairs upstream of rs1175550 (Table 1, Supplementary Figs S1 and S2). We found no significant correlations for the remaining six variants (Supplementary Figs S1 and S2). The two variants showed near-perfect linkage disequilibrium (LD), with rs1175550G being linked to rs143702418CGCA in 149 out of 150 samples (Table 1 and Supplementary Data S1). Furthermore, in the three predominant *SMIM1* intron 2 alleles (comprising 96% of all identified alleles) in Europeans in the 1000 Genomes project the rs1175550G/rs143702418CGCA and rs1175550A/rs143702418C genotypes were always linked (Supplementary Fig. S3). Thus, both rs1175550 and rs143702418 associate with *SMIM1* and Vel antigen expression, yet the strong LD precluded statistical separation of their effects using samples from individuals of European ancestry.

### rs1175550 and rs143702418 independently influence SMIM1 and Vel antigen expression

Hypothesising that the LD between rs1175550 and rs143702418 might be different in other populations, we examined the repertoire of *SMIM1* intron 2 alleles in the 1000 Genomes catalog. We observed 32 different alleles, although nine of these were only present in a single individual in 1000 Genomes, and could therefore represent sequencing artifacts. Analysis of alleles detected in at least two individuals showed that these cluster mainly by rs1175550 genotype, indicating that this variant appeared evolutionarily before the other variants in the region (Supplementary Fig. S4).

**Figure 2:**
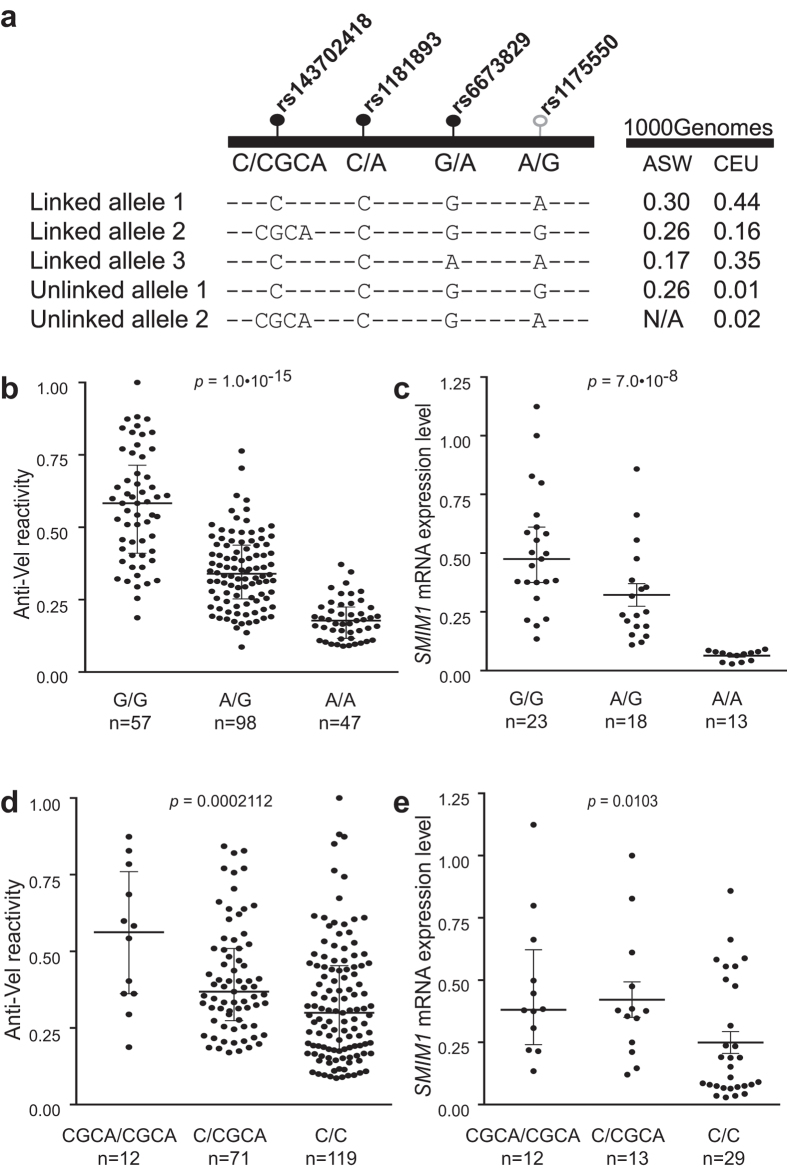
Deconvolution of the effects of rs1175550 and rs143702418. (**a**) The five most common *SMIM1* intron 2 alleles between rs143702418 and rs1175550 (complete list of alleles in Supplementary Fig. S3) along with their frequencies in the 1000 Genomes catalog (populations CEU, Utah Residents of Northern and Western European Ancestry; ASW, Americans of African Ancestry in South-Western USA). (**b**) Vel antigen expression on RBCs of the African American samples as measured by flow cytometry with human anti-Vel serum and PE-conjugated goat-anti-human secondary antibody (MFI, median fluorescence intensity relative to the sample with the highest expression), grouped according to rs1175550 genotype Values from all samples were plotted with indication of the median and IQR. Statistical analyses were performed with Kruskal-Wallis one-way analysis of variance with Dunn’s multiple comparisons test. (**c**) Expression of *SMIM1* mRNA in African American samples (RT-qPCR using the 2^−ΔΔCT^ method with normalisation against the sample with the highest expression), grouped according to rs1175550 genotype. Values from all samples were plotted with indication of the median and IQR. Statistical analyses were performed with Kruskal-Wallis one-way analysis of variance with Dunn’s multiple comparisons test. (**d**) Grouping of values in (**b**) according to rs143702418 genotype. (**e**) Grouping of values in (**c**) according to rs143702418 genotype. Association data for the six other SNPs in the region are given in Supplementary Figs S5 and S6.

Analysing the 1000 Genomes data, we noted a difference in genotype frequency between the two variants in the African super population (allele frequency 0.60 and 0.26 for rs1175550G and rs143702418CGCA, respectively), meaning the two variants are not in tight LD: the frequency of the unlinked rs1175550G/rs143702418C allele was 0.26 in African Americans, compared to 0.01 in Europeans (Fig. 2a). To deconvolve the effects of rs1175550 and rs143702418, and thereby assess their causality, we analysed samples from 202 African American blood donors. These samples showed a broader repertoire of *SMIM1* intron 2 alleles when compared to the Swedish samples, including alleles where the linkage between rs1175550 and rs143702418 was broken (Supplementary Data S2).

Genotype frequencies in the African American sample set matched those in the 1000 Genomes catalog (Table 1). In this sample set, we found rs1175550 to be associated with *SMIM1* mRNA and Vel antigen expression (*p* = 1.0∙10^−15^ for Vel antigen expression; *p* = 7.0∙10^−8^ for *SMIM1* mRNA), whereas rs143702418 showed weaker association (*p* = 0.0002112 for *SMIM1* mRNA; *p* = 0.0103 for Vel antigen expression) (Fig. 2b–e). None of the other candidate variants showed any significant correlation (Supplementary Figs S5 and S6).

**Figure 3:**
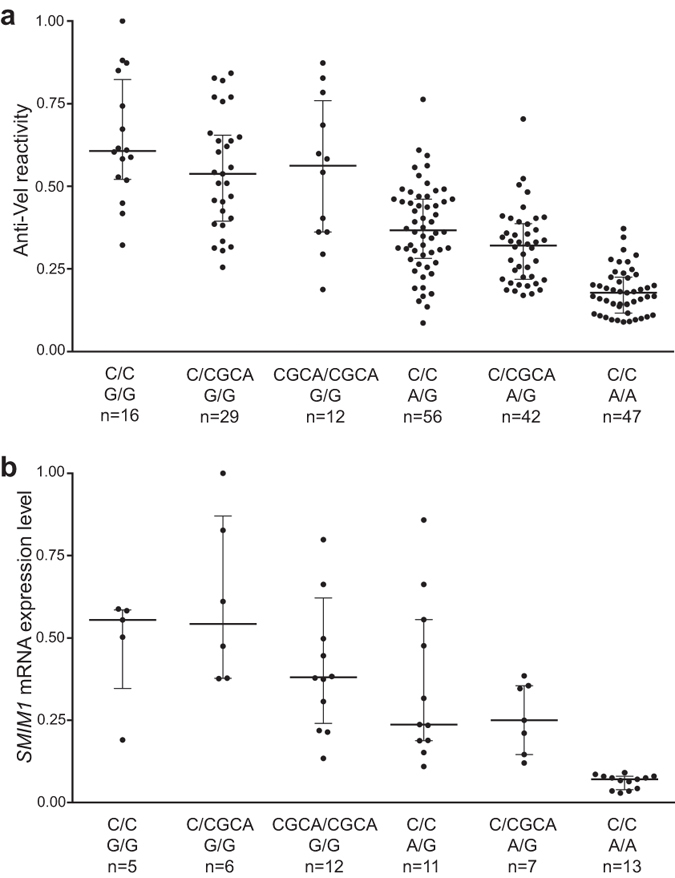
Conditional analysis indicates that rs143702418 has a small effect on *SMIM1* expression. Data from the African American collection were grouped by rs143702418-rs1175550 haplotype. The three genotypes for each of the two variants give a total of nine combinations, however, since the haplotype rs43702418CGCA:rs1175550A was not present in any of the samples, only six possible combinations are depicted here. (**a**) Flow cytometry data. All samples plotted individually with indication of the median and IQR. (**b**) RT-qPCR data. All samples plotted individually with indication of the median and IQR.

To test if the association with rs143702418 represents an independent effect, we used multiple linear regression including both rs1175550 and rs143702418 in the model. We found that samples heterozygous for rs143702418 had significantly lower Vel antigen expression (effect estimate, *p* = 0.00398) compared to homozygosity for rs143702418C (Fig. 3a), and observed a trend towards further decrease for samples homozygous for rs143702418CGCA (effect estimate, *p* = 0.08939). This was consistent with an increase in the model *R*^*2*^ as compared to a model including rs1175550 alone (adjusted *R*^*2*^ = 0.5237 vs. 0.5064 for rs1175550 alone; *p* < 2.2∙10^−16^ for model), indicating that rs143702418 has a small but independent effect (Fig. 3a, Supplementary Table S1). This trend was mirrored by the mRNA expression levels, although it was not statistically significant, possibly due to the limited sample size (Fig. 3b). No independent effects were observed for the other six candidate SNPs (Supplementary Table S1).

### rs1175550 and rs143702418 are transcriptionally active

Sequence analysis showed that rs1175550 and rs143702418 are located at predicted binding sites for erythroid transcription factors. For rs1175550, the major allele predicts a non-canonical GATA-1 site, GATT, modified by the minor allele to GGTT. For rs143702418, we observed that the major allele comprises a core motif for KLF1 (CACCC), which is modified by the minor allele to CACGCACC.

**Figure 4:**
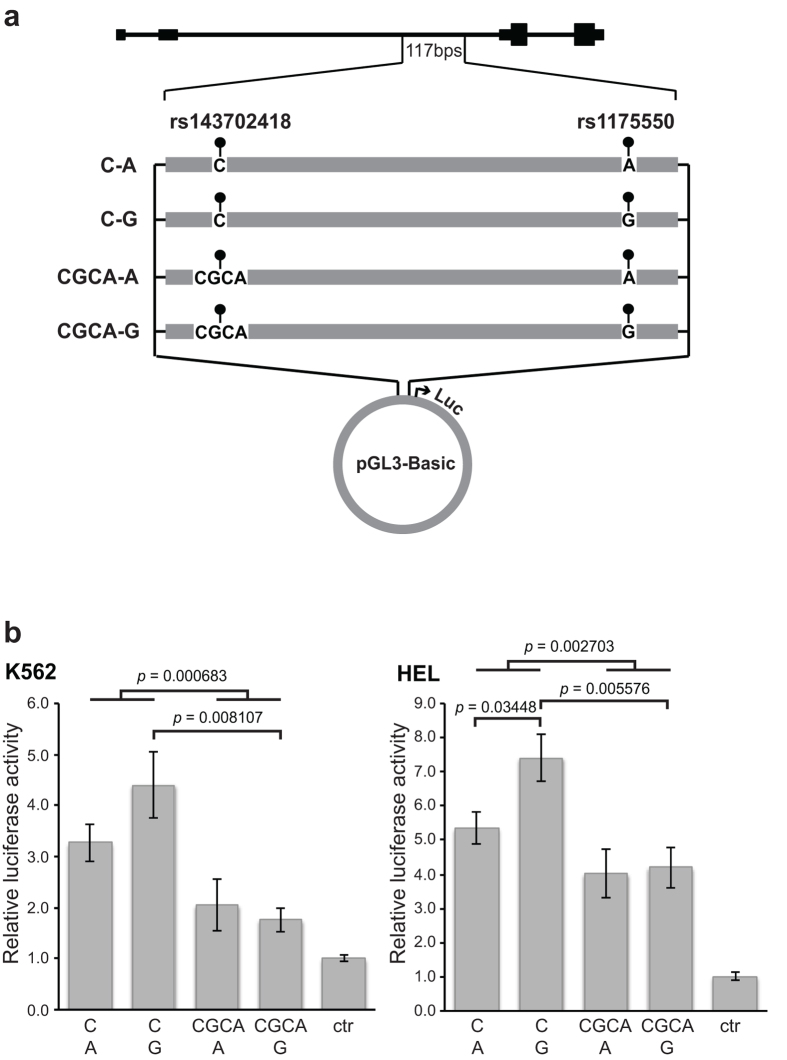
rs1175550 and rs143702418 independently increase luciferase activity. Four DNA fragments of *SMIM1* intron 2 (full sequences in Supplementary Table S2) corresponding to all four possible alleles of the two biallelic variants were inserted upstream of the luciferase gene in the pGL3-Basic vector and assayed for luciferase activity in K562 (**b**, left panel) and HEL cells (**b**, right panel). Values are fireflly/renilla ratios normalised to pGL3-Basic without insert (ctr), plotted as mean ± SEM and based on two different experiments performed in triplicate (n = 6) for each cell line. Pairwise, two-tailed unpaired t-tests were performed on all groups; selected *p*-values less than 0.05 are indicated. All groups were significantly different from the pGL3-Basic control vector.

**Figure 5:**
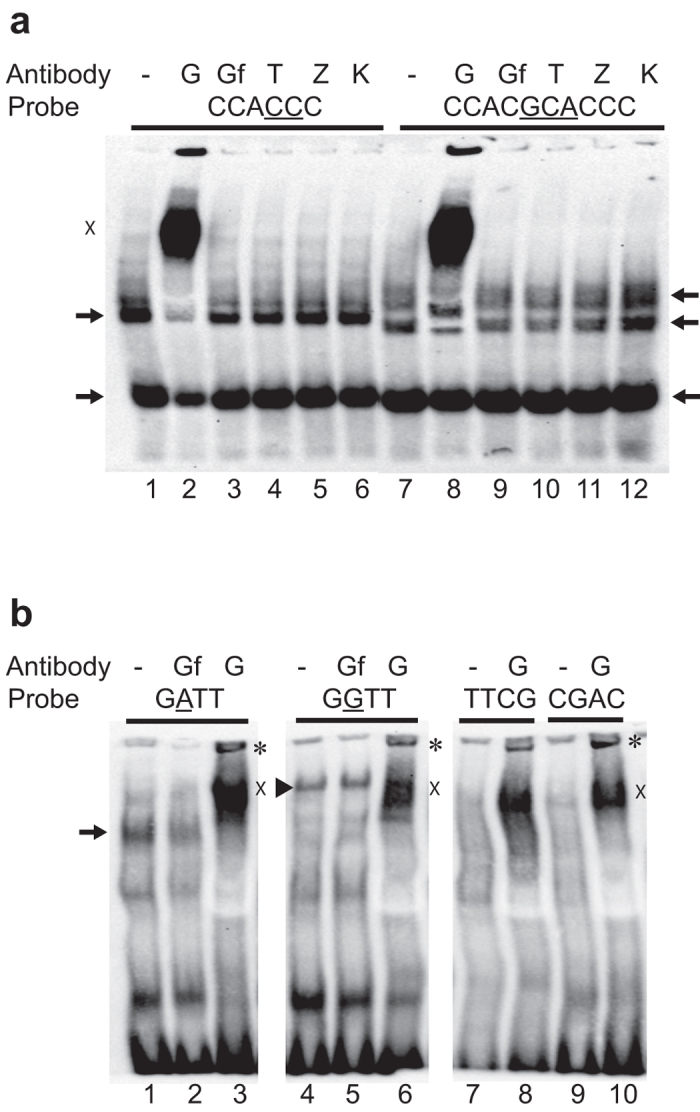
Gel shift analyses reveal binding of different protein complexes to either allele of both SNPs. Nuclear extracts from K562 cells were incubated with biotin-labelled probes spanning either allele of the two variants with or without antibodies. In the panels, arrows denote shifts by unknown proteins binding to the labelled probes; asterisks denote potential protein complexes supershifted by antibodies. X denotes an unspecific smear seen with anti-GATA-1 in all experiments; abbreviations G: Anti-GATA-1; Gf: Anti-Gfi-1B; T: Anti-TAL1; Z: Anti-ZBTB7A; K: Anti-KLF1. (**a**) Different binding patterns were seen with probes against the two rs143702418 alleles (rs143702418C and –CGCA). Antibodies were added to test for potential supershift reactions where indicated. (**b**) Different binding patterns were also seen with probes against the two rs1175550 alleles. Antibodies for GATA-1 and Gfi-1B were added for supershift reactions where indicated. (Super-) Shifts were also performed with probes where the central AG[A/G]TTG sequence was mutated (lanes 7–10). Arrowhead denotes the specific complex shifted only by the rs1175550G probe.

For functional validation of rs1175550 and rs143702418, we made reporter constructs corresponding to the four theoretical alleles containing the combinations of these two biallelic variants (Fig. 4a). Consistent with our association data, luciferase assays in K562 and HEL erythroleukemia cells showed higher activity with both rs1175550G and rs143702418C (Fig. 4b), and both constructs with rs143702418C showed higher activity than those with rs143702418CGCA (K562 *p* = 0.000683; HEL *p* = 0.002703). The highest luciferase activity was found for the construct also carrying the rs1175550G (*p* = 0.03448 in the HEL cells). Gel shift assays with nuclear extracts from K562 cells showed distinct binding patterns with probes mapping to the minor and major alleles for both rs143702418 and rs1175550 (Fig. 5a,b), most notably a distinct, shifted band only seen with the rs1175550G probe (Fig. 5b, lane 4). These results further support that the two variants independently modulate *SMIM1* and Vel antigen expression, although rs1175550 has a more powerful effect.

### Identification of TAL1 as a candidate factor underlying the increased SMIM1 expression associated with rs1175550G

Based on ChIP-seq data from ENCODE (Fig. 1a), we identified GATA-1, KLF1, TAL1 and ZBTB7A as candidate factors for regulating *SMIM1* expression. These factors have been previously identified as erythroid, and their temporal expression profiles in erythrocyte development follow that of *SMIM1* (Supplementary Fig. S7)^10^. We also included Gfi-1B as its binding motif AATC (reverse-complement GATT) matched the sequence at rs1175550, and it has been shown to associate with GATA-1 and mediate transcriptional repression^11^,^12^,^13^.

We carried out gel shift analyses with antibodies to the selected transcription factors. Firstly, we observed a supershift with anti-GATA-1 for rs1175550 (Fig. 5b, lanes 3,6). However, this signal was not allele-specific and additional analyses with probes mutated at the predicted non-canonical GATA-1 site at rs1175550 yielded similar results, indicating that GATA-1 does not bind directly to rs1175550 (Fig. 5b, lanes 8,10). Secondly, with anti-Gfi-1B, we observed that the strong band seen with both rs1175550A and rs1175550G probes was weakened with anti-Gfi-1B, whereas the allele-specific band observed only with the rs117550G probe remained unaffected (Fig. 5a, lanes 3,9). These data indicate that GATA-1 and Gfi-1B bind near rs1175550 (directly or indirectly) regardless of genotype, making it unlikely that the increased expression associated with rs1175550G is explained by altered binding of GATA-1 or Gfi-1B.

**Figure 6:**
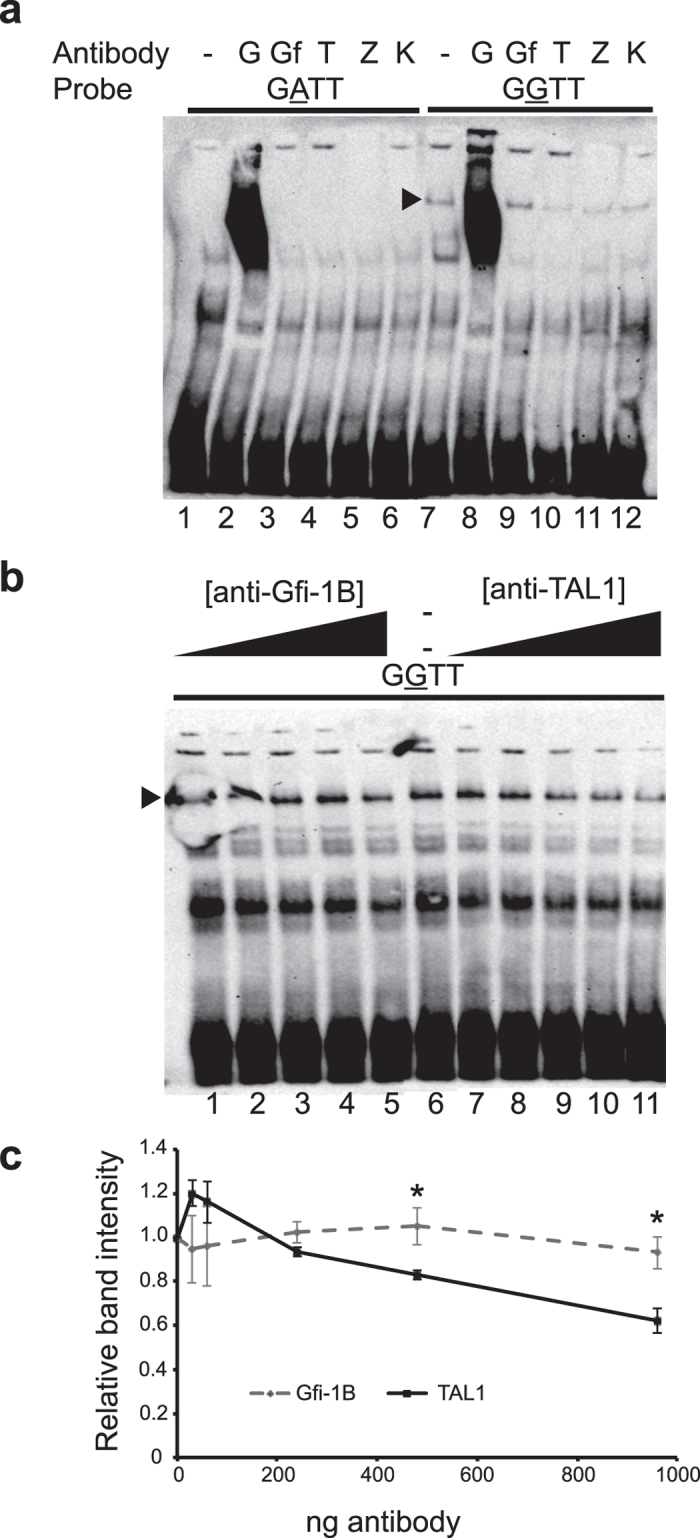
Identification of TAL1 as part of the complex binding exclusively to rs1175550G. Nuclear extracts from K562 cells were incubated with biotin-labelled probes spanning either allele of rs1175550. Arrowheads denotes the specific complex shifted only by the rs1175550G probe; (**a**) EMSA supershift reaction with the two alleles of rs11175550 and five antibodies. Disappearance of shifted bands when antibody is added indicates a competitive effect for the same spot on the probe. Antibody abbreviations, G: Anti-GATA-1; Gf: Anti-Gfi-1B; T: Anti-TAL1; Z: Anti-ZBTB7A; K: Anti-KLF1. (**b**) EMSA supershift reaction with the rs1175550G probe and increasing amounts of anti-Gfi-1B and anti-TAL1. (**c**) The diminishing rs1175550G-specific bands in (**b**) were quantified by densitometry analysis and plotted relative to the test condition with no antibody added (lane 6). Data is presented as means of three independent experiments with error bars indicating ± standard error of the mean. One-tailed, unpaired Student’s T-tests were performed pairwise for each antibody concentration. *p-values equal 0.03022 and 0.01293, respectively.

In contrast to the non-allele-specific reactions seen with anti-GATA-1 and anti-Gfi-1B, we achieved suppression of the rs1175550G-specific signal with anti-TAL1 (Fig. 6a, lane 10). No similar effects were seen with anti-Gfi-1B, anti-ZBTB7A or anti-KLF1 (Fig. 6a), while the TAL1 effect was dose-dependent (Fig. 6b,c). This is particularly interesting since only three bps upstream of the rs1175550 is a near-perfect match for the E-box motif CAGNTG, which is a known binding site for TAL1 in heterodimer complexes with E12 and E47^14^. Finally, we observed no allele-specific supershift or suppression for rs143702418 using these antibodies (Fig. 5a). In summary, our results suggest that differential binding of TAL1, or a TAL1-containing complex, could mediate the increase in *SMIM1* and Vel antigen expression associated with rs1175550G.

## Discussion

The variation in antigen expression among Vel+ individuals is clinically important, as it may lead to erroneous typing of Vel+ blood as Vel− with anti-Vel sera^15^,^16^. While rs1175550 zygosity may explain some of this variation, the causality of this sequence variant has been unclear. Fine-mapping the genomic neighbourhood of rs1175550, a regulatory region in *SMIM1* intron 2, we discovered that the previously unreported trinucleotide insertion rs143702418 also correlates with *SMIM1* and Vel antigen expression. While the effects of rs1175550 and rs143702418 were inseparable in Swedish samples, deconvolution in African American samples revealed that rs1175550 and rs143702418 both modulate Vel expression, although rs1175550 has the strongest effect. Luciferase and gel shift assays supported that both variants are transcriptionally active.

Although the exact mechanisms remain to be elucidated, we identified increased binding of TAL1 as a potential explanation for the increased SMIM1 expression associated with rs1175550G. This is in concordance with recently published data suggesting that TAL1 preferentially binds to rs1175550G^17^. Since the discussed TAL1 and GATA-1/Gfi-1B binding motifs are only three base pairs apart, one could speculate that steric hindrance does not allow these factors to bind simultaneously and that the A > G substitution favours TAL1 binding.

In conclusion, our findings provide novel insight into the regulation of *SMIM1* and the Vel blood group antigen, and provide further reason to take rs1175550 and rs143702148 into account when evaluating the correlation between Vel blood group phenotype and genotype. Insight into what governs blood group expression levels on erythroid cells can be important for our understanding of host-pathogen interactions since many blood group molecules serve as involuntary receptors for microbial agents and may therefore act as susceptibility markers for disease. Even if no such role has yet been proven for SMIM1, it has been hypothesised to be a long-sought malaria receptor^1^.

Recently, trans-ancestry association analysis has been proposed as a way to refine associations between genetic variants, quantitative traits and human diseases, yet only a few examples have been published so far^18^,^19^,^20^. Here we exploited pre-existing population-based data sets and trans-ancestry association analysis to deconvolve highly correlated effects. We predict that these approaches will be increasingly important for identifying the molecular-genetic effects of GWAS loci.

## Methods

### Blood samples

We used anonymised, peripheral blood samples from 150 Swedish blood donors (routine donations at Clinical Immunology and Transfusion Medicine, Lund, Sweden), and 202 self-declared African-American blood donors (routine donations at New York Blood Center, New York, NY, USA). No donors were approached solely for the purpose of this study. Genomic DNA and total RNA were isolated using standard methods. Samples were screened for the 17-bp deletion in *SMIM1* exon 3^1^ and carriers were excluded.

### Sanger sequencing

For Sanger sequencing of the 500-bp candidate regulatory region (hg38 coordinates chr1:3,774,672–3,775,171), we used the following primers: forward primer 1: 5′-TCTGTAGGGCCTCCCCAA-3′, forward primer 2: 5′-ACAGGGACCGGTCTAGCTGTA-3′, reverse primer 1: 5′-CTCCTAACAGCAGCCTCAGAG-3′ and reverse primer 2: 5′-TGTTAAGTCAGGCGACAGACC-3′. PCR products were sequenced using the BigDye Terminator v3.1 Cycle Sequencing kit on an ABI 3500 Dx Genetic Analyzer (Applied Biosystems). Additional Sanger sequencing was done at GATC Biotech, Germany. For peak calling and base determination we used CodonCode Aligner v4.1 software (CodonCode Corporation, USA).

### Reverse transcription quantitative PCR

Total RNA was extracted by Trizol LS purification (Ambion) and converted to cDNA using High-Capacity cDNA Reverse Transcription Kit (Applied Biosystems). Quantitative PCR was carried out on cDNA using an assay mix specific for *SMIM1* (Applied Biosystems assay no. Hs01369635_g1). *SMIM1* expression was quantified as 2^−ΔΔCT^ against the sample having the highest expression value. For normalisation across multiple qPCR runs these samples were included in each experiment.

### Flow cytometry

Red blood cells (RBCs) were incubated with human polyclonal anti-Vel followed by PE-conjugated F (ab′)2 Fragment Goat Anti-Human IgG (Jackson ImmunoResearch, #109–116–098) as described previously^1^. Cells were analysed on a FACS Calibur flow cytometer using CellQuest v3.3 and FACS Diva v6.1.3 software (Becton Dickinson). Values were taken as median fluorescence intensity (MFI) and all samples were normalised against the sample with the highest value in either collection.

### Western blot analysis

RBC membranes were prepared as described previously^1^, and analysed using Any kD Mini-PROTEAN TGX Stain-Free gels and the V3 Western Workflow (BioRad Laboratories). Following transfer, PVDF membranes were incubated with rabbit polyclonal anti-SMIM1 followed by horseradish peroxidase (HRP)-labelled goat anti-rabbit IgG (Agrisera, Sweden). Bands were visualised on ChemiDoc Touch (BioRad Laboratories) and analysed using ImageLab software v5.2 (BioRad Laboratories).

### Luciferase assay

Four DNA fragments of 117 bp or 120 bp (synthesised by Integrated DNA Technologies, USA) corresponding to the possible combinations created by the two variants rs143702418C/CGCA and rs1175550A/G (Supplementary Table S2) were inserted upstream of the luciferase gene in the pGL3-Basic luciferase vector (Promega) using restriction enzymes *Nhe*I and *Hind*III. DH5α competent *E. coli* cells were transformed with each construct, correct insertion was confirmed by sequencing and positive clones were amplified and purified with Plasmid Midi Kit (Qiagen).

K562 and HEL cells were cultured at 37 °C and 5% CO_2_ in RPMI 1640 medium (Gibco, Life Technologies) supplemented with 10% fetal bovine serum (Gibco). 2∙10^6^ cells were mixed with 2 μg construct and 0.2 μg Renilla luciferase construct and electroporated at 960 μFD and 280 V. Following incubation for 24 hours, dual luciferase assays were performed according to manufacturer’s protocol (Dual-Luciferase Reporter Assay System, Promega). All experiments were performed in triplicate.

### Electrophoretic mobility shift assay

Nuclear proteins were extracted from human K562 erythroleukemia cells as described^21^ and gel shifts were performed as described previously^22^. We used the following double-stranded probes (variant underlined): for rs143702418: 5′-GCCCTGCCCCACCCCCCCCTCCC-3′ and 5′-GCCCTGCCCCACGCACCCCCCCTCCC-3′, and for rs1175550: 5′-CTGCAGCCTAGATTGGGCCACAA-3′ and 5′-CTGCAGCCTAGGTTGGGCCACAA-3′. Additionally, we used two “scrambled” probes with the region surrounding the rs1175550 variant mutated (underlined): 5′-CTGCAGCCTCTTCGGGGCCACAA-3′ and 5′-CTGCAGCCTCCGACGGGCCACAA-3′. All probes were biotin-labeled at the 5′ end of both strands, unlabeled competitor probes with identical sequences were used to test for specificity. Supershift assays were performed using anti-GATA-1 (ActiveMotif, #39319), anti-Gfi-1B (B-7, Santa Cruz Biotechnology), anti-KLF1 (H-210, Santa Cruz Biotechnology), anti-LRF (ZBTB7A, H-6, Santa Cruz Biotechnology) and anti-TAL1 (ActiveMotif, BTL-73) antibodies. 2 μg antibody was added to the reaction mix and incubated 1 hour on ice, before addition of labeled probes, which in turn incubated 30 minutes at room temperature.

### Statistical analysis

Kruskal-Wallis one-way analysis of variance with Dunn’s multiple comparison tests as well as multiple linear regression models were used to test for association between genotypes vs. *SMIM1* (RT-qPCR) or Vel antigen expression (flow cytometry). In a first analysis, we included only rs1175550 as the explanatory variable. To assess the effect of variants conditioned on rs1175550, we carried out additional analyses using bivariate models with each variant together with rs1175550. For the phylogenetic analysis, we used phased genotyping data from The 1000 Genomes Project, phase 3 for the studied region in *SMIM1*. To reconstruct the phylogeny, we calculated the Euclidean distance between the different alleles, and clustered the alleles using the neighbour-joining method^23^. R version 3.2.2, Graphpad Prism version 7.0a and Microsoft Excel were used for data analyses.

## Additional Information

**How to cite this article**: Christophersen, M. K. *et al*. *SMIM1* variants rs1175550 and rs143702418 independently modulate Vel blood group antigen expression. *Sci. Rep.* **7**, 40451; doi: 10.1038/srep40451 (2017).

**Publisher's note:** Springer Nature remains neutral with regard to jurisdictional claims in published maps and institutional affiliations.

## Supplementary Material

Supplementary Information

Supplementary Data S1

Supplementary Data S2

## Figures and Tables

**Table 1:** Genotype frequencies for the eight variants included in the study.

	rs1184341			rs2797432			rs143702418			1181893		
	C/C	C/T	T/T	G/G	A/G	A/A	C/C	C/CGCA	CGCA/ CGCA	C/C	A/C	A/A
Experimental, SWE^a^	0.69	0.12	0.19	0.35	0.40	0.25	0.57	0.36	0.07	0.97	0.03	0.00
1000 Genomes, CEU^b^	0.71	0.25	0.04	0.24	0.57	0.19	0.67	0.29	0.04	0.97	0.03	0.00
Experimental, AA^c^	0.44	0.04	0.16	0.75	0.21	0.04	0.59	0.35	0.06	0.98	0.02	0.00
1000 Genomes, ASW^d^	0.49	0.39	0.11	0.64	0.34	0.02	0.54	0.39	0.07	1.00	0.00	0.00
	**rs6673829**			**rs1175550**			**rs9424296**			**rs1175549**		
	**G/G**	**G/A**	**A/A**	**A/A**	**A/G**	**G/G**	**C/C**	**C/A**	**A/A**	**A/A**	**A/C**	**C/C**
Experimental, SWE	0.48	0.40	0.12	0.56	0.37	0.07	0.88	0.12	0.01	0.68	0.17	0.15
1000 Genomes, CEU	0.39	0.48	0.12	0.7	0.27	0.03	0.92	0.08	0.00	0.69	0.29	0.02
Experimental, AA	0.85	0.13	0.01	0.23	0.49	0.28	0.94	0.06	0.00	0.00	0.72	0.28
1000 Genomes, ASW	0.67	0.31	0.02	0.25	0.46	0.3	0.96	0.03	0.00	0.26	0.46	0.28

^a^Swedish collection.

^b^CEU: Utah Residents with Northern and Western European Ancestry.

^c^African American collection.

^d^ASW: Americans of African Ancestry in Southwest USA.
