# Supplementary material for: SMIM1 variants rs1175550 and rs143702418 independently modulate Vel blood group antigen expression
Source: Sci Rep. 2017 Jan 13;7:40451. doi: 10.1038/srep40451 (PMC5233989; doi:10.1038/srep40451)
Supplement: Supplementary Information [file srep40451-s1.doc]

**SUPPLEMENTARY INFORMATION**

***SMIM1* variants rs1175550 and rs143702418 independently modulate Vel blood group antigen expression**

Mikael K. Christophersen^1^, Magnus Jöud^1,2^, Ram Ajore^1^, Sunitha Vege^3^, Klara W. Ljungdahl^1^, Connie M. Westhoff^3^, Martin L. Olsson^1,2^, Jill R. Storry^1,2,*^, Björn Nilsson^1,*^

^1^Division of Hematology and Transfusion Medicine, Department of Laboratory Medicine, Lund University, Lund, Sweden. ^2^Clinical Immunology and Transfusion Medicine, Laboratory Medicine, Office of Medical Services, Lund, Sweden. ^3^Laboratory of Immunohematology and Genomics, New York Blood Center, New York City, NY, USA.

^*^These authors contributed equally to the manuscript.

**Contents**

**Page 3** Supplementary Figure S1 – Flow cytometry data of the Swedish collection clustered according to individual SNP genotypes.

**Page 5** Supplementary Figure S2 – QPCR data of the Swedish collection clustered according to individual SNP genotypes.

**Page 7** Supplementary Figure S3 - Haplotypes between rs143702418 and rs1175550 in the 1000 Genomes catalog

**Page 9** Supplementary Figure S4 – Phylogenetic tree of all SNPs in the region, based on the 1000 Genomes catalog

**Page 11** Supplementary Figure S5 – Flow cytometry data of the African American collection clustered according to individual SNP genotypes.

**Page 13** Supplementary Figure S6 – QPCR data of the African American collection clustered according to individual SNP genotypes.

**Page 15** Supplementary Figure S7 – Temporal expression of erythroid genes in CD34+ cells cultured towards erythropoiesis.

**Page 17** Supplementary Table S1. Conditional correlation analyses using multiple linear regression on SNPs in the African American collection.

**Page 18** Supplementary Table S2. DNA fragments inserted into pGL3-Basic vector and used for luciferase assays.

**Page 19** References.

**Supplementary Figure S1**

Median Fluorescence Intensity values of erythrocytes from samples in the Swedish collection grouped according to genotypes of the remaining seven SNPs in the *SMIM1* intron 2 regulatory region. All samples are relative to the sample in the collection having the highest value. All values are represented with the median and interquartile range (IQR) indicated. p-values were calculated using Kruskal-Wallis one-way analysis of variance with Dunn’s multiple comparisons test (three groups) or Mann-Whitney test (two groups).

# Supplementary Figure S2

Relative quantification values of *SMIM1* mRNA levels from samples in the Swedish collection grouped according to genotypes of the remaining seven SNPs in the *SMIM1* intron 2 region. All samples are relative to the sample in the collection having the highest value, which was included in each RT-qPCR run. All values are represented with the median and IQR indicated. p-values were calculated using Kruskal-Wallis one-way analysis of variance with Dunn’s multiple comparisons test (three groups) or Mann-Whitney test (two groups).

# Supplementary Figure S3

All haplotype combinations existing between four of the variants covered in the study – rs143702418, rs1181893, rs6673829 and rs11175550. Data was obtained by analysing individual genotypes of the 1000 Genomes catalog phase 3 (2,504 samples). Of specific note is the high prevalence of the unlinked allele (red) among Africans. Alleles are grouped into five superpopulations African, Ad Mixed American, Eastern Asian, European and South Asian. The populations ASW (Americans of African American descent in Southwest USA) and CEU (Utah Residents with Northern and Western European Ancestry) are subgroups of the African and European superpopulations, respectively.

**Supplementary Figure S4**

Phylogenetic tree of the prevalent alleles in the regulatory region with more than 2 representations in the 1000 Genomes catalog, phase 3. Bold font indicates the variants included in this study. Superpopulations are AFR: African, AMR: Ad Mixed American, EAS: Eastern Asian, EUR: European, SAS: South Asian.

**Supplementary Figure S5**

Median Fluorescence Intensity values of erythrocytes from samples in the African American collection grouped according to genotypes of the remaining six SNPs in the SMIM1 intron 2 region. All samples are relative to the sample in the collection having the highest value. All values are represented with the median and IQR indicated. p-values were calculated using Kruskal-Wallis one-way analysis of variance with Dunn’s multiple comparisons test (three groups) or Mann-Whitney test (two groups).

**Supplementary Figure S6**

Relative quantification values of *SMIM1* mRNA levels from samples in the African American collection grouped according to genotypes of the remaining six SNPs in the *SMIM1* intron 2 region. All samples are relative to the sample in the collection having the highest value, which was included in each RT-qPCR run. All values are represented with the median and IQR indicated. p-values were calculated using Kruskal-Wallis one-way analysis of variance with Dunn’s multiple comparisons test (three groups) or Mann-Whitney test (two groups).

# Supplementary Figure S7

Temporal expression values of selected genes in adult CD34+ hematopoietic progenitor cells cultured towards erythrocytes^1^. Cells were grown for eleven days in serum-free medium containing erythropoietin, interleukin 3 and stem cell factor and array-profiled for gene expression every other day. Dataset is available in NCBI Gene Expression Omnibus, accession number GSE4655. Datapoints are means of three independent experiments, error bars indicate ± SD.

**Supplementary** Table S1

Conditional correlation analysis. Multiple linear regression models were constructed with rs1175550 and each of the other seven variants included individually.

**Supplementary Tabel S2**

DNA fragments inserted into pGL3-Basic vector and used in luciferase assays. Six random basepairs were incorporated in either end to facilitate restriction enzyme digestion together with unique restriction enzyme sites (italic) for *Nhe*I (5’ end) and *Hind*III (3’ end), and the *SMIM1* genomic sequence (hg38 coordinates chr1:3,774,859-3,774,987) between and flanking the two variants, rs143702418C/CGCA and rs1175550A/G (underlined).

**C-A**

TTCGGA*GCTAGC*GCCCTGCCCCACCCCCCCCTCCCCTGGCCAGCCTCCCCAGAG

GCCAGAAGGCGCCTTATCGGGCAGGGTTAAGGAGGGGGACAGTTATCAGGGGCT

GCAGCCTAGATTGGGCCACAA*AAGCTT*TTCGGA

**C-G**

TTCGGA*GCTAGC*GCCCTGCCCCACCCCCCCCTCCCCTGGCCAGCCTCCCCAGAG

GCCAGAAGGCGCCTTATCGGGCAGGGTTAAGGAGGGGGACAGTTATCAGGGGCT

GCAGCCTAGGTTGGGCCACAA*AAGCTT*TTCGGA

**CGCA-A**

TTCGGA*GCTAGC*GCCCTGCCCCACGCACCCCCCCTCCCCTGGCCAGCCTCCCCA

GAGGCCAGAAGGCGCCTTATCGGGCAGGGTTAAGGAGGGGGACAGTTATCAGGG

GCTGCAGCCTAGATTGGGCCACAA*AAGCTT*TTCGGA

**CGCA-G**

TTCGGA*GCTAGC*GCCCTGCCCCACGCACCCCCCCTCCCCTGGCCAGCCTCCCCA

GAGGCCAGAAGGCGCCTTATCGGGCAGGGTTAAGGAGGGGGACAGTTATCAGGG

GCTGCAGCCTAGGTTGGGCCACAA*AAGCTT*TTCGGA

**References**

1 Keller, M. *A. et a*l. Transcriptional regulatory network analysis of developing human erythroid progenitors reveals patterns of coregulation and potential transcriptional regulators*. Physiol Genomi*c**s** 28, 114-128, doi:10.1152/physiolgenomics.00055.2006 (2006).
